# Identification of deleterious non-synonymous single nucleotide polymorphisms using sequence-derived information

**DOI:** 10.1186/1471-2105-9-297

**Published:** 2008-06-27

**Authors:** Jing Hu, Changhui Yan

**Affiliations:** 1 Department of Computer Science, Utah State University, Logan, UT 84322, USA

## Abstract

**Background:**

As the number of non-synonymous single nucleotide polymorphisms (nsSNPs), also known as single amino acid polymorphisms (SAPs), increases rapidly, computational methods that can distinguish disease-causing SAPs from neutral SAPs are needed. Many methods have been developed to distinguish disease-causing SAPs based on both structural and sequence features of the mutation point. One limitation of these methods is that they are not applicable to the cases where protein structures are not available. In this study, we explore the feasibility of classifying SAPs into disease-causing and neutral mutations using only information derived from protein sequence.

**Results:**

We compiled a set of 686 features that were derived from protein sequence. For each feature, the distance between the wild-type residue and mutant-type residue was computed. Then a greedy approach was used to select the features that were useful for the classification of SAPs. 10 features were selected. Using the selected features, a decision tree method can achieve 82.6% overall accuracy with 0.607 Matthews Correlation Coefficient (MCC) in cross-validation. When tested on an independent set that was not seen by the method during the training and feature selection, the decision tree method achieves 82.6% overall accuracy with 0.604 MCC. We also evaluated the proposed method on all SAPs obtained from the Swiss-Prot, the method achieves 0.42 MCC with 73.2% overall accuracy. This method allows users to make reliable predictions when protein structures are not available. Different from previous studies, in which only a small set of features were arbitrarily chosen and considered, here we used an automated method to systematically discover useful features from a large set of features well-annotated in public databases.

**Conclusion:**

The proposed method is a useful tool for the classification of SAPs, especially, when the structure of the protein is not available.

## Background

It is estimated that around 90% of human genetic variations are differences in single bases of DNA, known as single nucleotide polymorphisms (SNPs) [1]. Among them, non-synonymous single nucleotide polymorphisms (nsSNPs), also known as single amino acid polymorphism (SAPs), that cause amino acid changes in proteins have the potential to affect both protein structure and protein function [2]. Some of the mutations in SAP sites are not associated with any changes in phenotype and are considered functional neutral, but others bringing deleterious effects to protein function and are responsible for many human genetic diseases [3,4]. Recent years have seen an explosion in the number of SAPs in public databases, such as dbSNP [5], HGVBASE [6] and SWISSPROT [7]. The large size of these databases presents a challenging hurdle for annotating the effects of all SAPs by experimental approaches. Therefore, computational methods that can quickly distinguish diseasing-causing SAPs from neutral SAPs are in urgent need.

Many methods have been proposed to classify SAPs. Earlier methods are based on empirical rules [8,9] or probabilistic models [10]. Recent methods are based on machine learning techniques, such as decision trees [11,12], random forests [13], neural networks [14,15], support vector machines [12,13,16-18]s. Some of these methods [9,15,16] explore only information derived from protein sequence. Others use both structural and sequence features of the SAP sites. A limitation of using structural features as input is that the methods are not applicable when protein structures are unknown. Additionally, all previous methods only consider a small set of features arbitrarily chosen. Systematic analysis is still needed to identify features that play vital roles in determining the effects of SAPs.

In this study, we explored the feasibility of classifying SAPs using only information derived from protein sequence. We compiled a set of 686 features based on previous studies and AAindex [19], a public database of amino acid properties. These features are independent of the structure of SAP sites. Thus, the developed method can be used to distinguish disease-causing SAPs from neutral SAPs even when the structures of SAP sites are not known. Then, we used a greedy method to discover useful features from the feature set. Using the 10 selected features, a decision tree method can classify SAPs with 82.6% accuracy and 0.607 Matthews Correlation Coefficient (MCC).

## Results

### Classification performance in cross-validation and independent test

Four subsets were used to select features using a greedy approach as described in Materials and Methods, and 10 features were obtained. These features were used to build predictors for classifying SAPs.

We first evaluated the proposed method using cross-validation. A four-fold cross-validation was performed on the four subsets that were used in feature selection. The results (Table 1) show that the method achieves 82.6% accuracy with 0.607 MCC.

**Table 1:** Performance of the proposed method

	Cross-validation	Independent test	Swiss-Prot^1^
MCC	0.607	0.604	0.42
Accuracy (%)	82.6	82.6	73.2
Sensitivity (%) (TP/(TP+FN))	94.9	94.7	84.0
True Positive Rate (%) (TP/(TP+FP))	81.6	81.6	75.0

^1^The proposed method was evaluated on SAPs from Swiss-Prot.

We then evaluated the proposed method using an independent test, in which the classifier was trained using the four subsets and then tested on an independent set. Note that the independent set was not seen by the algorithm during the feature-selection stage and the training of the classifier. The results (Table 1) show that the method achieves 82.6% accuracy with 0.604 MCC in the independent test. Table 1 also shows that the proposed method achieves consistent results in cross-validation and independent test.

To evaluate the usefulness of the feature-selection step, we used all the 686 features to build a predictor and then evaluated it using cross-validation. The predictor built using 686 features only achieved 0.503 MCC with 77.7% accuracy. Thus, the feature-selection step increased MCC by 0.104 and accuracy by 4.9%.

### Contributions of the selected features

**Figure 1:**
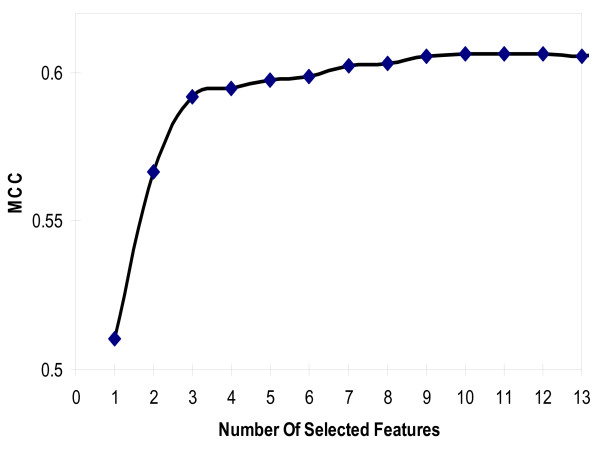
**Classification performance is improved as the feature selection progresses**. MCC (Matthew's Correlation Coefficient) increases as the number of selected features increases and reaches its maximum when 10 features are selected. The MCC remains unchanged when 11^th ^and 12^th ^features are selected. When more than 12 features are selected, the MCC slightly decreases.

In each step of the feature selection, the feature that brought the largest improvement in performance was chosen. Table 2 shows the 10 selected features in the order that they were chosen. Among the 10 features, the three features shown in italic fonts (*is_HLA, metal_seq_neibor, modres_seq_neibor*) were obtained from the study of Ye et al [18], *nor_diff_freq *is the normalized frequency difference between mutant-type residue and wild-type residue as defined in Materials and Methods, the other features are defined based on entries from AAindex. Figure 1 shows how the classification performance was increased when features were chosen. Among the 10 selected features, 4 (*nor_diff_freq, DAYM780301, HENS920103 and NAKH900106*) are related to residue frequency and substitution, 3 (*FEND850101, ZHAC000105 and MIYS850103*) are related to structure and contact energy, 2 (*metal_seq_neibor *and *modres_seq_neibor*) indicates whether the SAP site is close to functional sites, and 1 (*is_HLA*) shows the family of the protein. The fact that *is_HLA *is selected as a useful feature probably suggests that different rules apply to different families of proteins.

**Table 2:** Selected features

Feature	Annotation
*is_HLA*	Whether the protein containing the SAP belongs to HLA family [18].
nor_diff_freq	Normalized difference between mutant-type residue frequency and wild-type residue frequency.
DAYM780301	Log odds matrix for 250 PAMs [27]. The value between two amino acids shows how often one amino acid replaces another one in evolution.
FEND850101	Structure-Genetic matrix [28]. This matrix takes into account of the structural similarities of amino acids and the genetic code.
ZHAC000105	Environment-dependent residue contact energies [29]. The residue contact energies in different structural environment.
HENS920103	BLOSUM80 substitution matrix [30]. The value between two amino acids is defined based on the log likelihood of one amino acid substitutes the other by chance in sequence alignment.
NAKH900106	Normalized composition from animal [31]. Normalized residue composition calculated from animal mitochondrial proteins.
*metal_seq_neibor*	The sequence distance between the SAP site and its nearest residue holding the functional site with Feature Key of METAL [18].
MIYS850103	Quasichemical energy of interactions in an average buried environment [32].
*modres_seq_neibor*	The sequence distance between the SAP site and its nearest residue holding the functional site with Feature Key of MOD_RES [18].

Below is a part of the decision tree built by the decision tree algorithm.

   If (nor_diff_freq≤ -0.96) then

      Disease

   Else if (DAYM780301≤ 3.4) then

      If (nor_diff_freq≤ -0.37) then

         Disease

      Else ...

   Else if (DAYM780301>3.4) and (DAYM780301≤ 3.84) then

      If (nor_diff_freq≤ -0.90) then

         Disease

      Else ...

   Else if (DAYM780301>3.84) then

      If (Metal_seq_neighbor≤ 29) then

         Disease

      Else then

         Polymorphism

Following are some of the rules derived from the decision tree:

      Rule 1: "If (nor_diff_freq≤ -0.96), then Disease"

      Rule 2: "If (DAYM780301≤ 3.4) and (nor_diff_freq≤ -0.37), then Disease"

      Rule 3: "If (3.4<DAYM780301≤ 3.84) and (nor_diff_freq≤ -0.90), then

   Disease"

      Rule 4: "If (DAYM780301>3.84) then

         If (Metal_seq_neighbor≤ 29) then

            Disease

         Else then

            Polymorphism"

Here, nor_diff_freq ($nor\_diff\_freq=\frac{mt\_freq-wt\_freq}{wt\_freq}$), is a measure of the frequency difference between the mutant type and wild type. A negative value means that the wild type has higher frequency on the SAP position than the mutant type does. The lower is the negative value, the higher is the frequency difference between the wild type and the mutant type. Rule 1 says "If (nor_diff_freq≤ -0.96) then Disease". This rule indicates that if the mutation is from a wild type that has high frequency on the mutation site to a mutant type that has low frequency and if the frequency difference is very high, then the mutation will cause diseases. This reflects the hypothesis that if the SAP causes dramatic changes in sequence and structural stabilities, then the SAP is likely to be deleterious. DAYM780301 is the log odds matrix of PAM 250. The value between two amino acids shows how often one amino acid replaces the other in evolution. The higher is the value, the more frequently one amino acid replaces the other. To facilitate the explanation, let's borrow the term of similarity. The matrix is viewed as a measure of evolutionary similarity between amino acids. Higher values correspond to higher evolutionary similarities between residues. The four rules can be interpreted as below:

• Rule 1 says " regardless of the similarity between the mutant type and wild type, if the mutant type has lower frequency than the wild type does at the mutation site, and if the difference between their frequencies is very high (≤ -0.96), then the mutation is disease-related."

• Rule 2 says "if the similarity between the mutant type and wild type is very low (≤ 3.4), then although the difference between their frequencies is not very high (only ≤ -0.37), the mutation is still disease-related."

• Compared with rule 2, Rule 3 says "if the similarity between the mutant type and wild type is at median levels (3.4<DAYM780301≤ 3.84), then the mutation is disease-related only if the frequency difference between the mutant and wild types is high (has to be ≤ -0.90)."

• Compared with rules 1, 2 and 3, Rule 4 shows that if the similarity between the mutant type and wild type is very high (DAYM780301>3.84), then the difference between their frequencies is no longer a crucial factor in determining the effect of the mutation"

Together, these rules reflect the biological knowledge that if a mutation causes little changes (in chemical and physical properties, structural stabilities or other properties) to the protein, then mutation is likely neutral. Otherwise, it is likely diseased-related. The frequency difference between the mutant type and the wild type has been shown to be a useful feature for classifying the effect of mutations in many studies. Here these rules suggest that, in addition to the frequency difference, one also need to take into account the similarities (evolutionary, geometrical, or other properties related) between the mutant type and wild type.

### Comparisons with previously published methods

As discussed by Baldi et al. [20], in a two-class classification problem, if the numbers of the two classes are not equal, MCC is a better measure than accuracy for evaluating the classification performance. Thus, we use MCC as the main measure in the comparison of different methods.

Ye et al [18] developed a support vector machine (SVM)-based method, SAPRED, that classifies SAPs using 60 structural and sequence-derived features. In the current study, we used the same dataset Ye et al used in their study. On the same dataset, SAPRED achieved 82.6% accuracy and 0.604 MCC, and our method achieve 82.6% accuracy and 0.607 MCC. While the performances of the two methods are comparable, the virtues of our method are two-fold: (1) our method requires only sequence-derived information as input, and thus are applicable to SAPs whose structures are not available; (2) our method is based on a decision tree algorithm that is simpler than the SVM used by SAPRED. During the training of a decision tree-based classifier, there are no parameters needed to be tuned. In contrast, the training of a SVM requires enormous efforts to search for optimal parameters (e.g. C and gamma) and takes a longer time. Compared with SVM, the additional benefit of the decision tree is that the decision tree produces rules that can be easily interpreted. In this study, we also tried SVM instead of decision tree, but no improvement was observed by switching to SVM. Note that, in their study, Ye et al. also presented a sequence-version of SAPRED that only required sequence-derived features as input. But the sequence-version of SAPRED achieved only 0.577 MCC, which is lower than that of our method.

**Figure 2:**
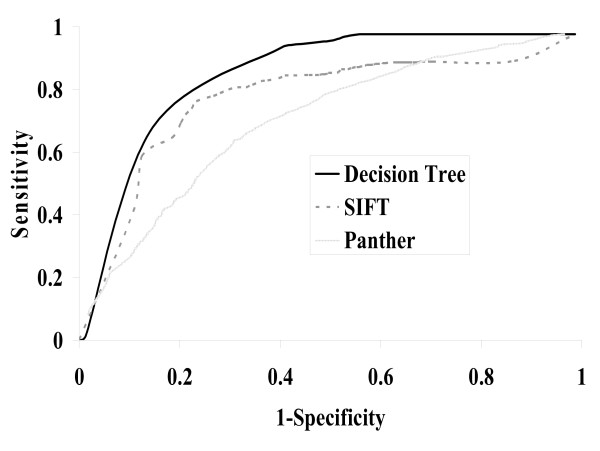
**Comparisons of the ROC curves of the proposed method, SIFT and Panther**. Area under ROC curve is 0.85 for Decision Tree, 0.77 for SIFT and 0.74 for Panther.

**Figure 3:**
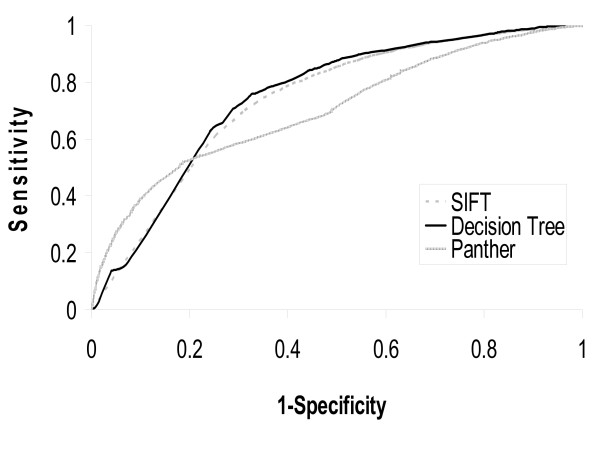
**ROC curves of the proposed method and others when tested on all SAPs from the Swiss-prot dataset**. Area under ROC curve is 0.75 for Decision Tree, 0.73 for SIFT and 0.70 for Panther.

Ye et al [18] also compared SAPRED with SIFT [21] using the same dataset used in the current study. Based on the results they reported, the method proposed in the current study achieves an increase of 0.127 in MCC over SIFT. We also submitted the dataset to Panther [22]. Panther only achieves 0.318 MCC. Comparisons of the ROC curves of SIFT, Panther and the current method confirm the improvement the current method over SIFT and Panther (Figure 2). As mentioned above, one merit of our method is that it can be applied to cases where the 3D structures of the proteins are not available. We also evaluated our method using all SAPs from the Swiss-Prot variant database. The proposed method achieves 0.42 MCC with 73.2% overall accuracy. In comparison, SIFT achieves only 0.33 MCC. Our method still outperforms SIFT. The ROC curve is showed in Figure 3.

Bromberg and Rost [15] developed a neural network method (SNAP) for classifying SAPs. They evaluated the method on a dataset obtained from Protein Mutant Database [23] and 78% accuracy was reported. The dataset from Protein Mutant Database is based on experimental amino acid substitutions, while the dataset used in this study is based on observed human alleles. Due to the difference in the datasets used in the two studies, we were unable to make a direct comparison between SNAP and the method proposed in the current study.

## Discussion

In contrast to previous studies that only considered a small set of arbitrarily chosen features, we used an automated method to discover features for distinguishing deleterious SAPs. The search space is more than ten times larger than those considered in previous studies. Based on the selected features, we developed a decision-tree based method for classifying SAPs. The proposed method only requires sequence-derived features as input. Thus, it can be applied to all SAPs. The performance of the proposed method is comparable to that of SAPRED, a state-of-the-art method that uses both structural and sequence-derived features as input, and is much higher than that of SIFT, a classic method for classifying SAPs.

## Conclusion

In conclusion, the proposed method is a useful tool for the classification of SAPs, especially, when the structure of the protein is not available.

## Methods

### Datasets

A dataset of SAPs was obtained from a recent study by Ye et al. [18]. It was collected from the variant pages of the Swiss-Prot knowledgebase. It has 3438 SAPs found in 522 proteins, including 2249 "Disease" and 1189 "Polymorphism" SAPs. We also evaluated the proposed method on the Humvar dataset from the PhD_SNP sever [16], which consists of all SAPs from the Swiss-Prot database. It has 12944 "Disease" and 8241 "polymorphisms" SAPs. The dataset is available online [24].

### Sequence attributes

#### A. Amino acid features obtained from AAindex

AAindex [19] is a database of numerical indices representing various physicochemical and biochemical properties of amino acids. Two types of entries are available in AAindex. The first type of entries has 20 values, with each value indicating the property of one amino acid. The second type of entries consists of a 20 × 20 matrix, giving the property between each pair of amino acids, e.g. substitution matrix. We downloaded the current version of AAindex (as of Sept 13, 2007), removed entries with missing values. 666 entries were left, with 531 from the first type and 135 from the second. For each entry *i*, we defined a feature for the SAP site that measured the distance between the wild-type residue and the mutant residue:

1) If entry *i *was an entry of the first type, then the feature was given by

$$f_{i}=\frac{index_{i}(mut)-index_{i}(wildtype)}{index_{i}(wildtype)}$$

where, index_i_(mut) and index_i_(wildtype) were the property values of wild-type and mutant residues given by entry *i*. Because some of the values in entry *i *could be 0, to avoid zero values in the denominator, the 20 values in entry i were normalized to the range of [0.1, 1.1].

2) If entry *i *was an entry of the second type, the feature was given by the value in the matrix corresponding to the pair of mutant and wild-type resides.

#### B. Sequence features used in previous studies

In their study, Ye et al. [18] calculated 60 structural and sequence attributes for each SAP site. Here, we took the features from their study and discarded those that were derived from the structure of the SAP sites. 19 features were left. One of them was residue frequency difference (diff_freq) between wild type and mutant type:

*diff *_*freq *= *mt *_*frq *- *wt *_ *freq*

where *mt_freq *and *wt_freq *are the frequencies of the mutant-type residue and the wild-type residue in the multiple alignment of homologous sequences. In this study, we introduced another feature (nor_diff_freq) by normalizing the frequency difference:

$$nor\_diff\_freq=\frac{mt\_freq-wt\_freq}{wt\_freq}$$

Finally, we obtained a set of 666+19+1 = 686 features for each SAP site. Note that in the calculation of these features for an SAP site, the structure of the SAP site was not required.

### Decision tree algorithm

Decision tree has been widely applied in many classification problems, including the classification of SAPs [11,12]. One benefit of using decision tree is that it generates classification rules that can be easily interpreted. In this study, we used the J48 decision tree algorithm implemented in WEKA [25].

### Performance measures

Let "Disease" be the positive class and "Polymorphism" be the negative class. Measures used in this study are defined below.

$$Accuracy=\frac{TP+TN}{TP+FN+TN+FP}$$

$$MCC=\frac{TP\times TN-FP\times FN}{\sqrt{\left(TP+FN)(TP+FP)(TN+FP)(TN+FN\right)}}$$

Where *TP *is the number of true positives (i.e., the number of "Disease" SAPs predicted as "Disease"); *TN *is the number of true negatives (i.e., the number of "Polymorphism" SAPs predicted as "Polymorphism"); *FN *is the number of false negatives (i.e., the number of "Disease" incorrectly predicted as "Polymorphism") and *FP *is the number of false positives (i.e., the number of "Polymorphism" incorrectly predicted as "Disease").

Accuracy is the overall percentage of SAPs correctly predicted. MCC (Matthews correlation coefficient) measures the correlation between predictions and actual class labels. In a two-class classification, if the numbers of the two classes are not equal, MCC is a better measure for evaluating the performance than accuracy [20]. In this study, the numbers of two classes ("Disease" and "Polymorphism") are not equal. Thus, MCC is used as the primary measure for evaluating the performance in this study.

### Feature selection, cross-validations, and independent test

We developed an automated approach to select useful features from the set of 686 features and applied a decision tree method to classify SAPs into "Disease" and "Polymorphism" classes. The proposed method was evaluated using both cross-validation and independent test. In the study of Ye et al. [18], the dataset was divided into five subsets at the protein level, such that SAPs from the same protein would be put into the same subset. This stringent criterion ensured more rigorous cross-validations than other studies. Thus, we used the same dataset partition as in Ye et al [18].

Four subsets were used to perform feature selection based on a (four-fold) cross-validation. In the four-fold cross-validation, there were four rounds of experiments. During each round of experiments, three subsets were used to train the classifier, and the remaining subset was used to test it. In each four-fold cross-validation, the same feature set was used in the four rounds of experiments. The average results of the four rounds were considered. The four-fold cross-validation was repeated using different feature sets (that were selected by the greedy approach mentioned below) until the optimal performance was reached.

The fifth subset (independent set) served as the test set in the independent test, in which the classifier was trained using the four subsets and then tested on the independent set. Note that the independent set was not seen by the algorithm during the feature-selection stage and the training of the classifier.

### Greedy approach for feature selection

Four subsets of SAPs were used to select features for building classifiers. Let *S *be the set of the selected features, *A *be the set of available features, and *N *be the size of *A*. At the beginning, *S *is empty and *N *= 686. Features were added into *S *using the following procedure:

(1) Pick one feature from *A*;

(2) Build classifiers using the newly picked feature and the features in *S*, and then evaluate the classifiers using a four-fold cross-validation;

(3) Repeat steps (1) and (2) *N *times, so that every feature in *A *is tried once. The feature that brings the biggest improvement in performance is removed from *A *and added into *S*.

This procedure continued until including more features into S does not increase the performance. In the end, 10 features were added into *S*.

## Availability and requirements

A web server based on the proposed method is available online [26].

## Authors' contributions

CY conceived of and designed the study, performed the analysis and drafted the manuscript. JH contributed to most of the computation. Both authors read and approved the final manuscript.
